# Optimal Dynamics for Quality Control in Spatially Distributed Mitochondrial Networks

**DOI:** 10.1371/journal.pcbi.1003108

**Published:** 2013-07-11

**Authors:** Pinkesh K. Patel, Orian Shirihai, Kerwyn Casey Huang

**Affiliations:** 1 Department of Bioengineering, Stanford University, Stanford, California, United States of America; 2 Boston University Medical School, Boston, Massachusetts, United States of America; 3 Department of Microbiology and Immunology, Stanford University, Stanford, California, United States of America; University of Notre Dame, United States of America

## Abstract

Recent imaging studies of mitochondrial dynamics have implicated a cycle of fusion, fission, and autophagy in the quality control of mitochondrial function by selectively increasing the membrane potential of some mitochondria at the expense of the turnover of others. This complex, dynamical system creates spatially distributed networks that are dependent on active transport along cytoskeletal networks and on protein import leading to biogenesis. To study the relative impacts of local interactions between neighboring mitochondria and their reorganization via transport, we have developed a spatiotemporal mathematical model encompassing all of these processes in which we focus on the dynamics of a health parameter meant to mimic the functional state of mitochondria. In agreement with previous models, we show that both autophagy and the generation of membrane potential asymmetry following a fusion/fission cycle are required for maintaining a healthy mitochondrial population. This health maintenance is affected by mitochondrial density and motility primarily through changes in the frequency of fusion events. Health is optimized when the selectivity thresholds for fusion and fission are matched, providing a mechanistic basis for the observed coupling of the two processes through the protein OPA1. We also demonstrate that the discreteness of the components exchanged during fusion is critical for quality control, and that the effects of limiting total amounts of autophagy and biogenesis have distinct consequences on health and population size, respectively. Taken together, our results show that several general principles emerge from the complexity of the quality control cycle that can be used to focus and interpret future experimental studies, and our modeling framework provides a road-map for deconstructing the functional importance of local interactions in communities of cells as well as organelles.

## Author Summary

Mitochondria are the powerhouses of eukaryotic cells, oxidizing glucose to produce ATP. Most cells harbor tens to hundreds of mitochondria in a constant state of flux, in which they fuse with one another, undergo fission, import proteins to grow larger, and eventually are recycled by autophagy. These dynamic processes depend on the electrical potential that is maintained across the mitochondrial inner membrane and powers the production of both ATP and detrimental reactive oxygen species. How do mitochondria maintain high membrane potential in the face of damage due to reactive oxygen species? Here, we develop a model to study how the reorganization of mitochondrial networks in space and time due to fusion, fission, and the experimentally observed development of membrane potential asymmetry after fission affect overall mitochondrial health. We show that health, which is a proxy for the mitochondrial membrane potential, is dominated by how density and motility affect the frequency of fusion events, and that several simple rules for the system kinetics lead to optimal quality control. This model predicts general behaviors that can be applied to specific studies of mitochondrial dynamics in a wide variety of cell types, and provides a framework for deconstructing complex organellar organization and their function in human disease.

## Introduction

All eukaryotic cells contain mitochondria, whose number and structure can vary substantially during the cell cycle [1] and between cell types [2]. One of the primary functions of mitochondria is the production of ATP through utilization of the electrochemical potential across the inner membrane (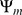) [3]. However, this process also produces reactive oxygen species (ROS), which can damage DNA and proteins [4]. The input-output relation between membrane potential and ATP production is highly nonlinear [5], [6], suggesting the importance of maintaining high membrane potential within an optimal range that results in high levels of ATP production with minimal ROS production. Indeed, mitochondria with low membrane potential are selectively degraded within autophagosomes [7].

Mitochondria are dynamic organelles that undergo frequent cycles of fusion and fission, leading to the formation of complex networks distributed heterogeneously across the cell [8]. The ultrastructure of the mitochondrial network varies significantly with cell type. Skeletal muscle cells contain long filamentous mitochondrial networks, while immune cells can contain large spherical mitochondrial aggregates [8], [9]. In normal rat kidney cells, mitochondria switch from a fragmented to a hyperfused state during the 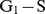 transition [1]. During periods of fusion, mitochondria exchange both soluble and membrane-bound components. The time scale of this exchange can vary significantly depending on the component being exchanged and its location in the mitochondrion; soluble matrix components mix rapidly (within seconds), while inner-membrane proteins mix on a time scale of tens of minutes [10].

Recent fluorescence microscopy studies have explored the relationship between 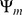 and mitochondrial fusion and fission in H9c2 cells, a subclone of a cell line derived from embryonic BD1X rat heart tissue that exhibits many of the properties of skeletal muscle [11], by simultaneously quantifying the levels of a 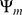-sensitive dye and of matrix-targeted photo-activateable green fluorescent protein (PA-GFP) [12]. By photo-activating the PA-GFP in only a small proportion of the mitochondria within a given cell, fusion events can be directly observed via the transfer of activated PA-GFP into a dark matrix compartment and the equilibration of 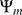. Fusion was observed to selectively involve mitochondria with high 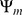 [12]. In addition, approximately 30% of fusion events were followed by a statistically significant difference in the membrane potentials of the two daughter mitochondria (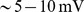); the mechanism behind the generation of this asymmetry in 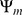 is currently unknown. Importantly, this difference has a large impact on the relative capacities for ATP and ROS production of the two daughter mitochondria [6].

Similar microscopy experiments on H9c2 cells revealed that the probability of fusion of two mitochondria in close contact was strongly dependent on whether one or both of the mitochondria were being actively transported at the time of contact [13]. This study also distinguished two different classes of fusion, one that allows complete mixing of matrix components and another that allows only partial mixing (transient fusion) [13]. This transient fusion usually occurs when two mitochondria on different microtubules come into contact, while complete fusion occurs when mitochondria are either stationary or if they are on the same microtubule [13]. This partial diffusion occurs mainly for membrane proteins, since diffusion of ions and soluble matrix proteins is very fast over the micron length scales of individual mitochondria, and suggests an absence of active mixing during fusion.

In support of the physiological importance of mitochondrial dynamics, the inhibition of fusion or fission leads to a deterioration of overall mitochondrial health in many organisms [14]–[16]. Deletion of mitochondrial fusion proteins Mfn1 and Mfn2 in mice is embryonically lethal, and the mitochondria in these mutant cells are fragmented and dysfunctional (depolarized with low ATP production) [16]. In the budding yeast *Saccharomyces cerevisiae*, deletion of the mitochondrial fusion protein FZO1 results in fragmentation of the mitochondrial network and loss of respiratory function [17]. A unifying hypothesis is that mitochondrial fusion-fission and autophagy form a quality-control axis that maintains mitochondrial health by segregating dysfunctional mitochondria into a separate pool of mitochondria that are unable to fuse, and undergo autophagy [14]. Recent evidence suggests that mitochondrial motility plays a key role in each of the steps associated with mitochondrial quality control, including fusion, fission, biogenesis, and lysosomal function. Motility is affected by both normal changes in the cell's physiological state (e.g., during the cell cycle) and in pathologies such as neurodegeneration [18], mutations in proteins such as the mitofusins [16] and Parkin [19], and calcium homeostasis pathologies [18]. Therefore, consideration of motility and the spatial distribution of the mitochondrial network is critical to address the effects of pathological changes on mitochondrial quality control.

A previous computational study of the effects of fusion, fission, and autophagy on mitochondrial health in the presence of ROS-mediated damage demonstrated that selective fusion and autophagy can result in stable average health of the mitochondrial population [20]. Moreover, post-fission asymmetry in 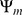 led to a further health enhancement, by using selective autophagy to ratchet against damage. The model was used to demonstrate that selective fusion could be important as an isolation mechanism that allows autophagy to recycle dysfunctional mitochondrial components. A different model of the effects of reducing mitochondrial fusion as the cell ages proposed two separate modes of mitochondrial damage [21]: stochastic damage similar to Ref. [20] and infectious damage, which was conjectured to be the main cause of the rapid depolarization observed after fission [12]. These studies were important first steps in modeling mitochondrial network dynamics. However, they did not consider the spatial distribution of mitochondria, preventing them from addressing whether cytoskeletal motility and mitochondrial density strongly affect quality control. Moreover, their artificial implementations of fusion in which all unfused mitochondria could interact, regardless of position, made it more difficult to connect model kinetics to experimental parameters.

Here, we present a computational model that incorporates the spatial dynamics of mitochondrial networks into the quality control cycle of the mitochondrial population. Similar to Ref. [20], we simplify the physiology of mitochondrial function to a health parameter mimicking 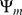. We show that the average health of the network is maximized when the fusion and autophagy processes have a common selective health threshold, suggesting a common mechanism of 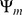-selectivity by the fusion and autophagy machinery. We also demonstrate that as long as the density of mitochondria is high enough to preserve a critical level of fusion, quality control is sufficient to maintain a healthy mitochondrial population. Moreover, the effect of any motion dependence of fusion can be understood simply as a shift in the effective fusion rate. In the absence of an active segregation mechanism, the molecular mechanism underlying the generation of asymmetry in 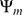 must be relatively discrete to benefit health, involving at most only a few tens of units that can be exchanged. Finally, we find that restricting the maximal rate of autophagy primarily affects the health of the network, while restricting mitochondrial replication (biogenesis) primarily affects the population size. Taken together, these results can guide the interpretation of experiments by revealing the processes for which cellular health has the greatest sensitivity.

## Results

### A model of stochastic, spatially dependent mitochondrial dynamics

**Figure 1:**
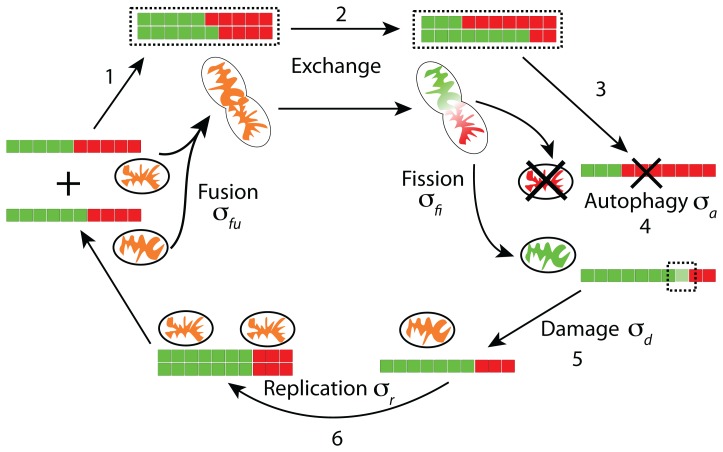
A cycle of fusion, fission, and autophagy contribute to quality control of mitochondrial health. Each mitochondrion is represented as a set of discrete health units that are in either a healthy (green) or damaged (red) state. (1) Fusion joins two (or more) mitochondria into a connected entity, though each mitochondrion is assumed to retain its original identity. Only mitochondria above a particular health threshold are permitted to fuse. (2) Fused mitochondria undergo stochastic exchange of a fixed number of health units. This exchange can lead to an asymmetry in the health of fused mitochondria, which occurs when these mitochondria undergo fission. (3) Fission separates fused mitochondria into isolated mitochondria or mitochondrial subnetworks. (4) Autophagy removes isolated mitochondria when their health is below the autophagy threshold. (5) Damage to mitochondrial health sets a health unit to the damaged state. (6) New mitochondria are produced via the cumulative effects of protein synthesis and import, and this replication event generates a copy of an existing mitochondrion that appears in the same physical neighborhood with the same number of health units as the progenitor mitochondrion. The variables representing the rates of each process (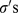) are shown next to their labels.

**Figure 2:**
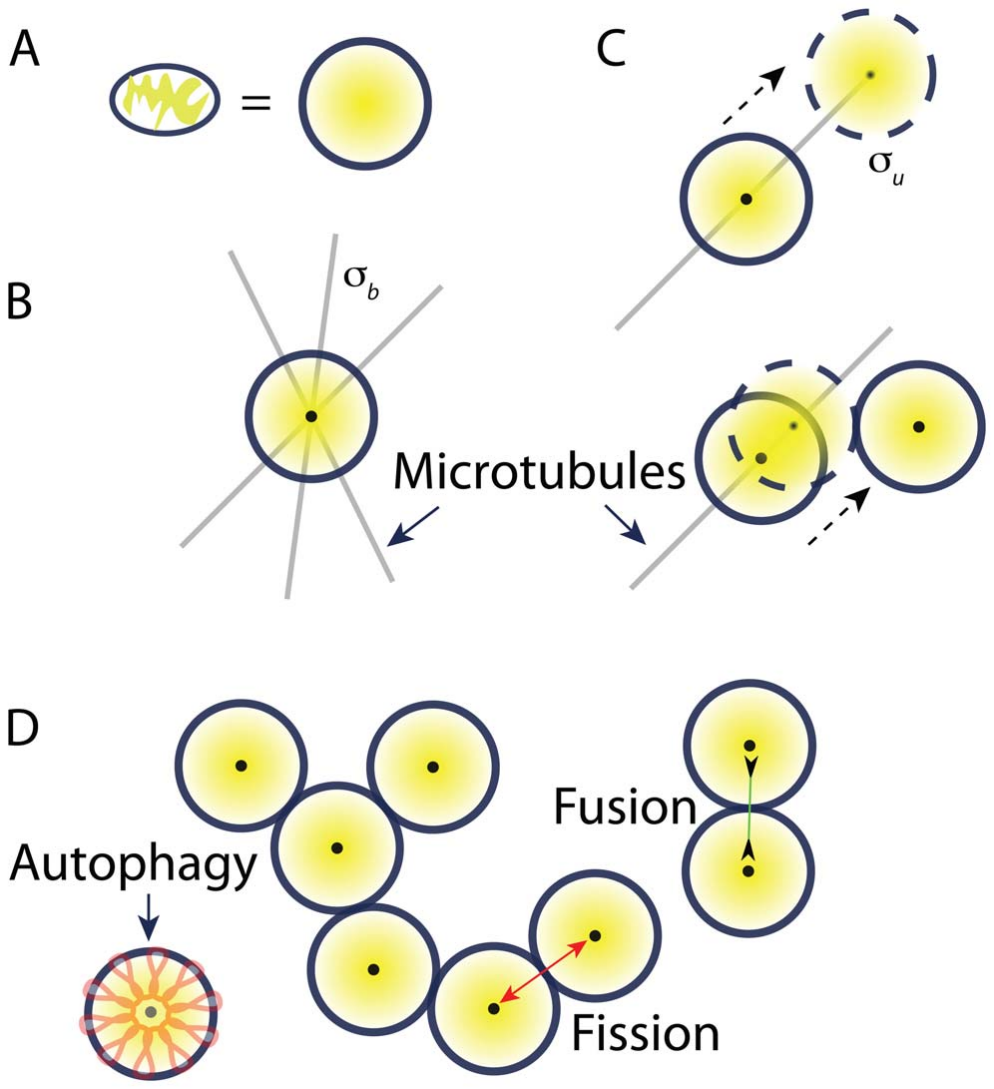
Active transport model of mitochondrial spatial dynamics. (A) We represent each mitochondrion as a circle with radius 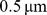. (B) Each stationary mitochondrion can bind a cytoskeletal filament pointed in a random direction at rate 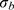. (C) A mitochondrion attached to a filament moves at constant velocity 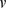 until it either unbinds at rate 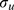 or encounters another mitochondrion or the edge of the cell. (D) In the presence of fusion and fission, this model produces mitochondrial networks with structures qualitatively similar to those observed *in vivo*. Autophagy of unfused mitochondria with poor health (orange) is critical for the establishment of functional mitochondrial networks, by allowing the replacement of dysfunctional mitochondria with healthy mitochondria that can still undergo fusion.

We encompass the diverse range of mitochondrial network properties by simulating the dynamics of mitochondria that undergo autophagy, replication, fusion, fission (Fig. 1), and transport along cytoskeletal filaments (Fig. 2). Each simulation represents a cell initialized with a given number of mitochondria (Methods). Similar to a previous study [20], we define a quantity for each mitochondrion that we will refer to as its “health,” which is a proxy for 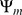. The ATP-producing potential of a mitochondrion is correlated with its 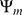 [5], [6]. When 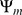 is below 180 mV; ATP production only becomes nonlinear under stress, when the mitochondrion is hyperpolarized and respiration is slowed [5]. Our simulations thus most closely mimic cells in unstressed conditions, when lower 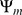 is directly correlated with lower ATP production.

Mitochondria lose their metabolic function over the course of days [22], although the specific cause of this loss is unclear. We treat the health of a mitochondrion as being composed of 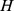 binary health units (HUs) that are either undamaged (1) or damaged (0); in each simulation, 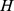 is fixed for all mitochondria. We model the effects of this damage on each HU as a Poisson process with constant rate 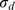 that switches an HU from undamaged to damaged (Fig. 1), mimicking the detrimental effects of ROS production on mitochondrial function. To determine the health of a given mitochondrion, we compute the sum of all the HUs and divide by the maximum number (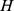). The limit of a continuous health quantity that mimics continuous 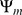 can in principle be realized by taking 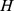 to be very large.

In our simulations, each mitochondrion behaves as an independent entity unless it is fused to another mitochondrion, in which case the fused mitochondria behave as one entity. Although fragmented mitochondria can have a distribution of sizes [23], we treat all networks as composed of individual mitochondria of a fixed size representing the average of this distribution. For computational simplicity, we represent each mitochondrion as a circle with radius 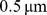 (Fig. 2A) [13]. Mitochondria are too large to diffuse in a densely packed cell at any appreciable rate, and consequently rely on active transport by molecular motors along cytoskeletal filaments [24]. In our model, we treat active transport as persistent diffusion along a dense microtubule network that allows travel in any direction (Fig. 2B). In our simulations, we restrict motion to only unfused mitochondria. Each unfused mitochondrion is treated as either stationary or attached to a cytoskeletal filament. An unfused mitochondrion can bind to a microtubule at rate 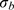 from anywhere in the cell, and the direction of motion is chosen at random from 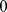 to 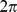 (Fig. 2B). An attached mitochondrion moves at a constant velocity until it detaches from the filament at rate 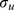 or until it contacts another mitochondrion, at which time fusion can occur (Fig. 2C, Methods). Upon fission, the daughter mitochondria again behave as independent entities unbound to a cytoskeletal filament.

We consider the mitochondria to be spatially distributed across a two-dimensional, square-shaped cell, a reasonable approximation of common experimental imaging conditions with cells spread on adhesive surfaces [25]. We incorporate the necessity for direct contact during fusion by determining a fusion probability for each pair of mitochondria based on whether they are touching (Methods). The explicit incorporation of mitochondrial proximity in fusion dynamics is a key feature of our model that distinguishes it from previous models [20], [21], and allows us to address experimental observations such as the dependence of fusion on intracellular mitochondrial transport. Selectivity is incorporated by only permitting fusion between mitochondria whose health exceeds a fusion threshold 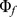. Fused mitochondria undergo fission at a fixed rate (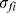) selected to reproduce the observed 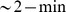 duration of fusion events in H9c2 cells [14]. In order to simulate the effects of 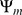 asymmetry after fissioning, we exchange a set number of randomly chosen HUs between the two fissioning mitochondria. This strategy simulates partial diffusion of contents between the fused mitochondria that is interrupted by fission. It also helps to partially retain the original mitochondrial identity, as observed in fluorescence microscopy experiments [12], [13], [26], [27]. In these “kiss and run” events, fused mitochondria retain their pre-fusion spatial extents post-fission despite large changes in their membrane potentials, suggesting that such partial diffusion along with retention of mitochondrial identity is a critical component of mitochondrial function.

During autophagy, mitochondria are engulfed by autophagosomes and delivered to lysosomes for lysis [7] (Fig. 2D). Autophagy is selective; only dysfunctional mitochondria with lower 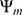 and hence lower ATP production potential are targeted to autophagosomes [7]. Therefore, we model autophagy by removing mitochondria whose health is less than a given autophagy threshold 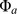 at a fixed rate 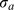, where we have assumed that the number of autophagosomes is not limiting for mitochondrial turnover. In accordance with experimental observations [12], we do not allow large mitochondria to undergo autophagy, since autophagosomes have a maximum size limit that we take to be a single, unfused mitochondrion.

The removal of mitochondria through autophagy is balanced by biogenesis, or replication [2]. Although increases in mitochondrial mass typically occur through the process of continuous growth followed by fissioning, for simplicity we model replication as discrete events that occur at rate 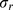 involving the instantaneous doubling in size and then fissioning of one mitochondrion into two (Methods). Given that the mitochondrial biomass (population size) in many cells is roughly constant [2], we assume that there is a metabolic limit to the replication rate in which replication asymptotically approaches zero as the population size 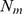 increases according to
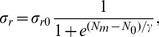
(1)where 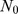 represents the typical number of mitochondria and 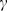 represents the variability in mitochondrial number. This implementation of autophagy and replication allows the mitochondrial number to vary with the kinetics of fusion, fission, and autophagy, unlike previous models that have imposed a fixed mitochondrial population size [20].

### Autophagy is required for maintaining a healthy mitochondrial population

In order to study how the mitochondrial population size and average health are affected by changes to the processes captured by our model (Fig. 1), we simulated the network dynamics within cells seeded with an initial population of 150 unfused mitochondria. In each simulation, the mitochondria were initially uniformly distributed within a two-dimensional cell whose geometry we approximate as square with sides 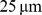. Except where indicated, each mitochondrion has 10 HUs, two of which are exchanged at the time of fission. The initial state of each HU was chosen randomly, giving mitochondria with a distribution of healths between 0 and 1 and an average across the population of 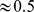.

**Figure 3:**
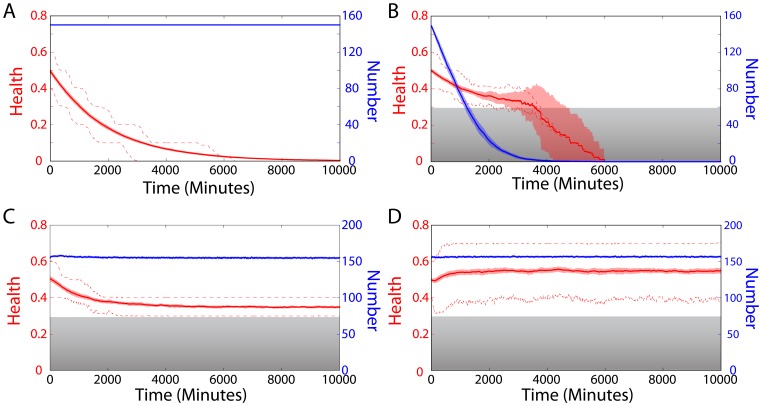
Maintenance of a healthy mitochondrial population requires autophagy and is enhanced by fusion and fission. (A) In the presence of damage alone, average mitochondrial health (red) decays exponentially. The dashed lines represent the 25th and 75th percentiles of health averaged over 25 independent simulations, while the shaded region represents one standard deviation above and below the average health across the simulations. The population size (blue) remains constant. (B) With the addition of autophagy, which removes mitochondria below an autophagy threshold of 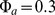 (gray region), the average steady-state health initially decreases more slowly than in (A), before the decreasing number of mitochondria reduces the population density to a level that makes it impossible to maintain health. The wide range of healths near this transition point is due to the small numbers of mitochondria per cell. (C) The addition of replication rescues the population size and health to a value just above the autophagy threshold. (D) The further inclusion of fusion, for mitochondria above a fusion threshold 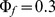, and fission increases the average steady-state health of the mitochondrial population. The variability in health increases due to the stochastic nature of the asymmetric exchange of HUs during fission.

Damage alone in the absence of fusion, fission, or autophagy resulted in an exponential decrease in the average mitochondrial health (Fig. 3A). The further inclusion of autophagy led to a monotonic decline in the total number of mitochondria in the cell (Fig. 3B). However, the average health decreased more slowly than in Fig. 3A, since autophagy selectively removed the unhealthy mitochondria from the cell. This decline in mitochondrial population size was counterbalanced by sufficiently high rates of biogenesis (Fig. 3C). In this case, the average steady-state health stabilized at a value close to the autophagy threshold 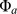.

**Figure 4:**
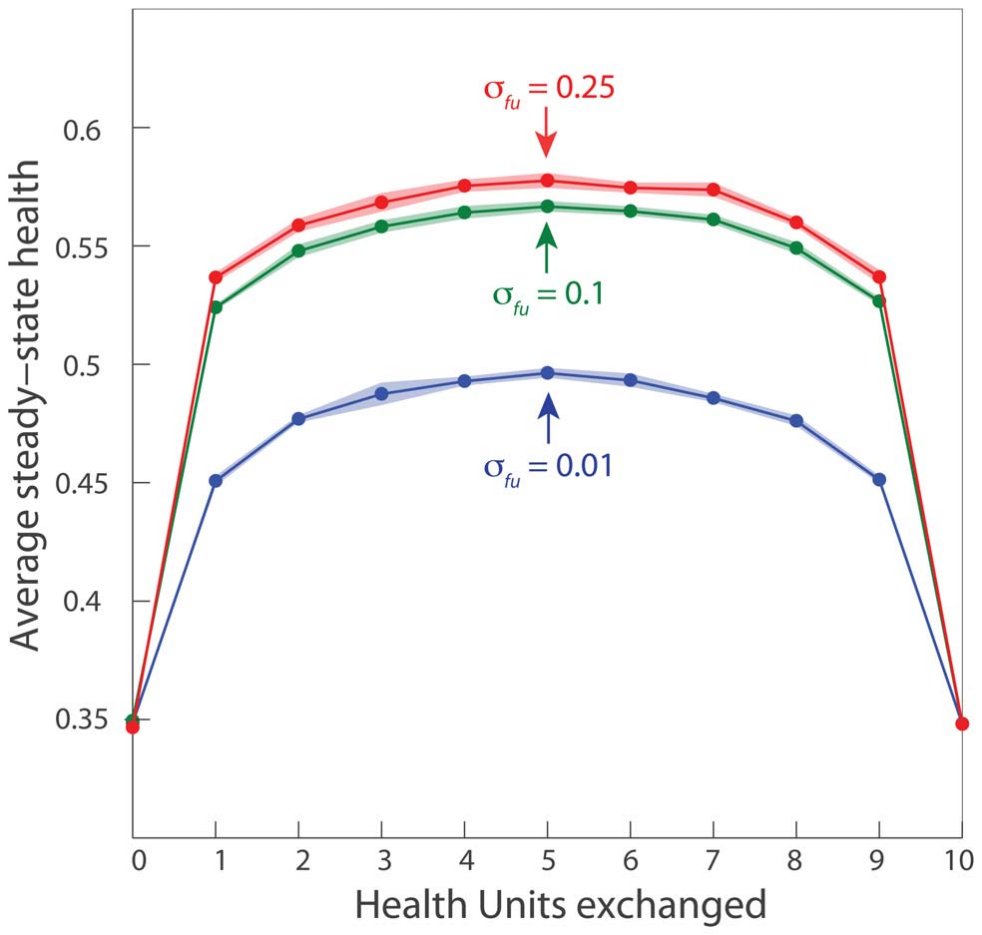
Maximal average steady-state health occurs for maximal asymmetry generation through stochastic exchange and fission. In each simulation, each mitochondrion has 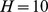 health units, and thus exchanging 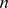 units is equivalent to exchanging 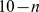 units. For all fusion rates, the average steady-state health is maximized when 5 units (or half the total number) are exchanged during each fusion and fission event. Health reaches a plateau for all values of exchanged HUs at fusion rates of 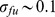. All fusion rates are in units of 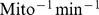.

As reported in Ref. [20], the average health can be substantially improved by selective fusion and asymmetric fission in conjunction with damage, autophagy, and replication (Fig. 3D). The exchange of HUs that occurs during fission stochastically leads to an asymmetry in the healths of the fissioning mitochondria. The probability of asymmetry development is highest when the two fused mitochondria have comparable healths, and when the number of HUs exchanged is half the total number 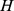 (Fig. 4). Randomly exchanging 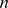 units is equivalent to exchanging 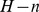 units since the identities of individual mitochondria can be switched without affecting the simulation. This dependence on exchanged units is similar across fusion rates, and the peak occurs at 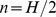 in all cases studied (Fig. 4).

**Figure 5:**
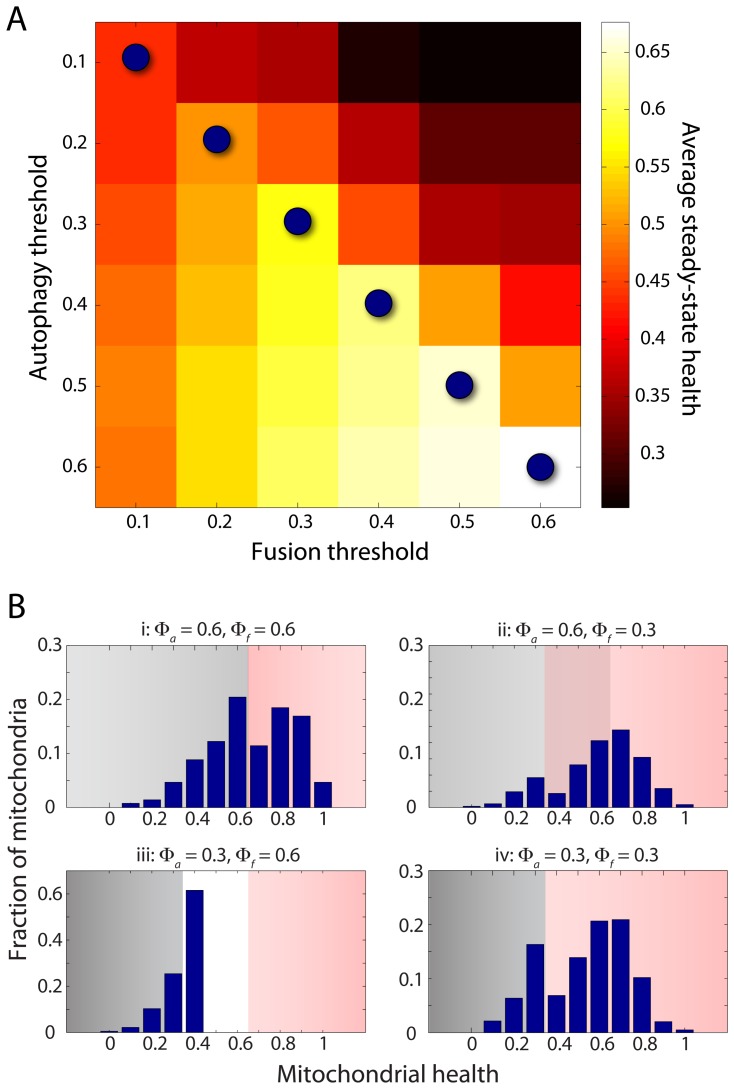
Mitochondrial health is maximized when the thresholds for autophagy (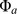) and fusion (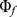) are equal. (A) For each 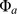, the average health (values represented by the color bar) is maximized when 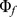 has the same value (blue circles), suggesting that the mechanism for fusion selectivity shares molecular components with autophagy selectivity. (B) Distributions of health across the mitochondrial population once simulations reach a steady state for different values of 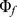 and 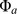, highlighting the effects of unequal thresholds (ii and iii). Gray regions indicate the mitochondria that can undergo autophagy, while red regions indicate the mitochondria that can undergo fusion.

Given that both fusion and autophagy have been observed to occur in a 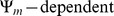 manner, we used our model to comprehensively determine the effects of 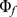 and 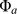 on health. For every value of 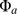, our simulations indicated that the steady-state health was maximized when 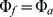 (Fig. 5A). When 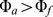, there is a large fraction of mitochondria with health between 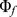 and 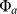 that are removed through autophagy even when they are still capable of fusion and hence improvement of their health could have occurred through ratcheting (Fig. 5Bii). When 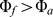, the consequences on health are more stark: the cell is unable to maintain mitochondria with health substantially above the autophagy threshold and hence they are incapable of fusion (Fig. 5iii). For thresholds above 0.6, the maximal average steady-state health was only slightly above the threshold values due to diminishing benefits from asymmetric exchange. Thus, for the remainder of our study, we set the two thresholds to be equal at 0.3 and studied other conditions that yielded optimal mitochondrial health.

### Health maintenance is insensitive to mitochondrial density above a critical level

**Figure 6:**
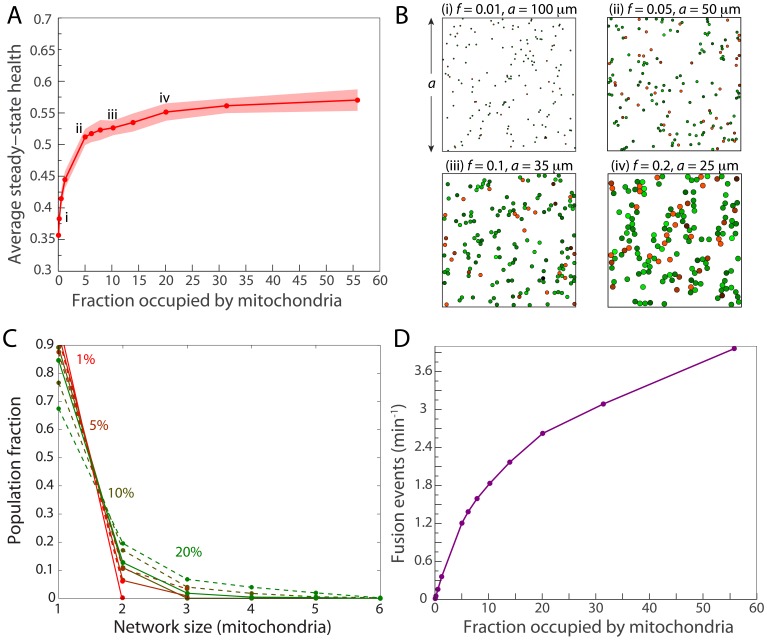
Health is insensitive to density as long as fusion events are sufficiently frequent. (A) In simulations within cells of different sizes, average steady-state health plateaus after a filling fraction of 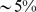. (B) Typical network architectures at the points labeled in (A). Each mitochondrion has a radius of 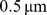 in all simulations and is colored according to its health. Mitochondria above and below 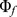 and 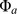 appear in shades of green and red, respectively. (C) Histograms of the number of mitochondria in networks of different sizes. The solid lines correspond to the networks formed only by fused neighbors, while the dashed lines correspond to networks of touching mitochondria regardless of fusion state. (D) The frequency of fusion events as a function of density, showing the same transition at 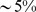 filling fraction.

To examine whether mitochondrial density affects the efficacy of quality control, we performed simulations in which we changed density by varying the size of the cell in order to maintain similar levels of stochasticity due to mitochondrial numbers in all cases. Interestingly, health began to saturate when the mitochondria occupied 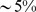 of the cell area, and the further increase was less than 10% when the filling fraction, 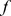, was increased to 55% (Fig. 6A). Typical network structures at each density exhibited a sharp transition around 5% from fragmented mitochondria to extended, fused networks (Fig. 6B,C), and this transition was closely coupled to a sharp increase in the number of fusion events per unit time (Fig. 6D). At low density, the mitochondria have a low probability of contacting each other, and hence are generally unable to exploit the benefits of fusion and fission before their health drops below 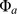. For the remainder of this study, we set the cell size to correspond to an intermediate filling fraction of 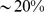, when health is relatively high and insensitive to changes in density.

### The motility dependence of fusion alters health primarily by modulating the overall frequency of fusion events

**Figure 7:**
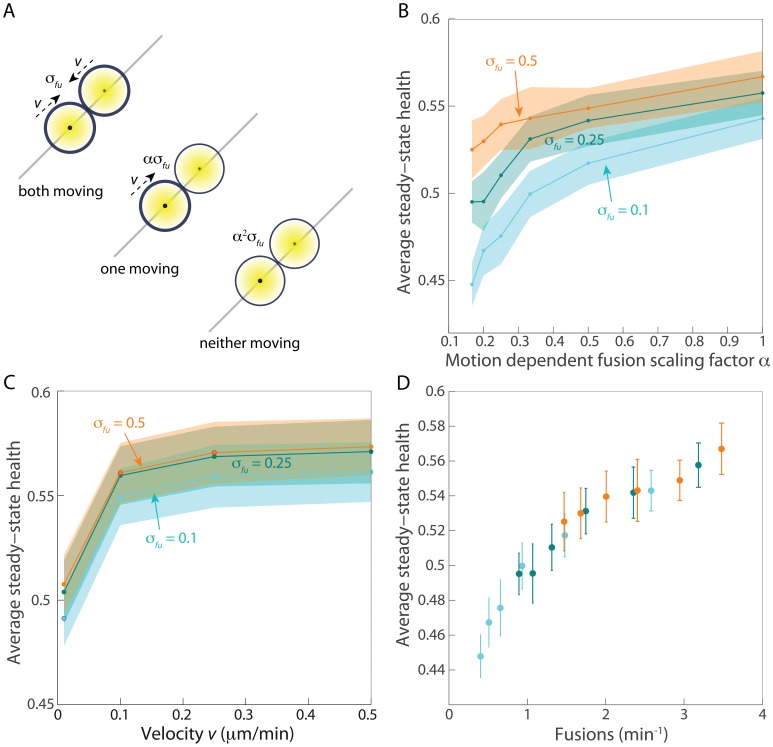
Motion dependence of fusion alters health by rescaling the effective fusion rate. (A) Rates of fusion were scaled down by 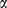 or 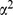 if only one or neither, respectively, of the fusing mitochondria were actively being transported at the time of fusion. Transport occurs at velocity 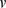. (B,C) Decreasing 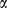 (B) or 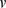 (C) for fixed 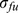 leads to a decrease in health. In (B), 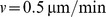; in (C), 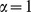. (D) Over a wide range of values of 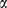 and 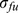, the frequency of fusion events was highly related to average health. The colors correspond to the same fusion rates specified in (B). All fusion rates are in units of 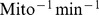.

Given experimental observations in H9c2 and rat insulinoma INS-1 cells that fusion is more likely to occur between mitochondria that are being actively transported along cytoskeletal filaments [13], we sought to determine whether this behavior would result in higher mitochondrial health compared with a uniform rate of fusion. While the purpose of intracellular mitochondrial transport is thought to be for supplying ATP on demand at locations such as neurotransmitter or hormone release sites, here we study the effects of such transport on quality control without considering other aspects of such motion related to spatially dependent ATP production.We scaled the fusion rate such that two proximal mitochondria would have a fusion rate of 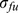, 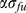, or 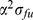 depending on whether both, one, or neither mitochondria were in motion at the time of fusion (Fig. 7A). We considered only values of 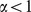, in which the fusion rate was decreased if one or both mitochondria were not moving. For the parameters in Fig. 3D (corresponding to 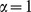), approximately 70%, 25%, and 5% of the fusion events resulted from these three classes of motility pairs, with an overall fusion frequency of 168 events/hr.

Lower values of 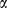 (more motion dependence) resulted in a decrease in health (Fig. 7B), though this was also coupled to a decrease in the frequency of fusion events and the degree of reduction in health was dependent on the fusion rate. Average health was relatively unaffected by a ten-fold decrease in the transport velocity 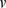, and then decreased at very slow velocities (Fig. 7C). We postulated that these decreases could be explained solely on the basis of the decrease in the effective fusion rate, and scanned a wide range of values of 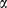 and 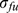. As hypothesized, the average steady-state health was highly related to the frequency of fusion events (Fig. 7D), demonstrating that fusion is the primary determinant of health maintenance, and that transport affects health through its indirect modulation of fusion frequency.

### Limiting the rates of autophagy and replication affects health and mitochondrial population size in distinct manners

**Figure 8:**
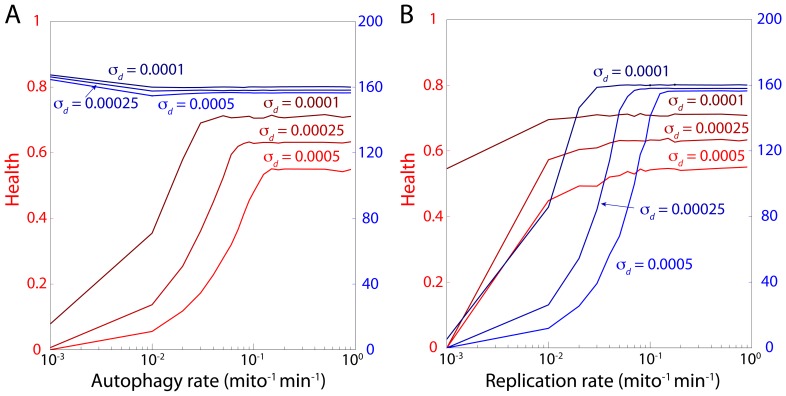
Maximal autophagy and replication rates affect health and mitochondrial population size in distinct manners. (A) Restricting the maximal autophagy rate causes a decrease in steady-state health with relatively little effect on population size, indicating that autophagy is critical to maintaining a healthy population. (B) In contrast, cells maintain high average health when the maximal replication rate is restricted, while the population size decreases. All damage rates are in units of 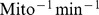.

Biogenesis and autophagy are limited by protein synthesis, import across the mitochondrial membranes, and the number of autophagosomes. To implement these constraints in our model, we enforced maximal rates of autophagy and replication (Methods). We observed that increasing the maximal autophagy rate from very low values exerted a large impact on average health (Fig. 8A), indicating that maintenance of autophagy above a critical level may be crucial for removing damaged mitochondrial components, thereby allowing biogenesis of less-damaged mitochondria and leading to a healthier mitochondrial population. Over a wide range of the maximal autophagy rate, the cell maintained a constant number of mitochondria, while the steady-state average health saturated at a value determined by the damage rate 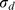 (Fig. 8A).

In contrast, a high average health was maintained over a wider range of maximum replication rates (Fig. 8B). However, decreasing the maximal replication rate reduced the size of the mitochondrial population. The transition from a stable to a decreasing population size occured in a range coincident with the sharp change in health due to caps on autophagy (Fig. 8A), illustrating the role of damage in constraining both health and population size when there are physiological limitations on autophagy and biogenesis.

### Discreteness is crucial for the effectiveness of membrane potential asymmetry

**Figure 9:**
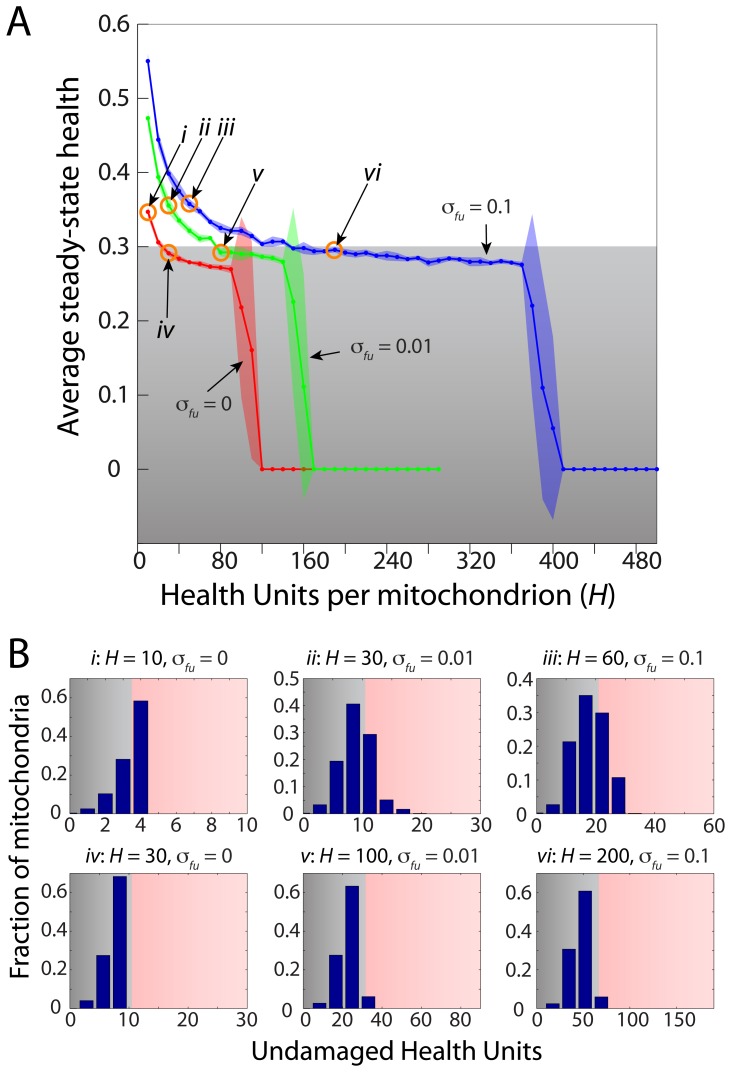
Maintenance of mitochondrial health requires a discrete health parameter. (A) The average steady-state health of the mitochondrial population can be maintained above the autophagy threshold when the number of HUs per mitochondrion is small, but falls off rapidly to zero due to loss of mitochondria via autophagy as the total number of HUs increases toward a continuum health variable. When the rate of fusion is nonzero, cells can tolerate less discreteness in the health parameter (more HUs) due to the additional stochasticity introduced by fission and by sequestering mitochondria from autophagy while fused. (B) Histograms of the healths of mitochondria in simulations conducted with different maximum HU values and fusion rates (points labeled 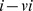 in (A)) show that the overall health depends on the distribution of mitochondria and this overall distribution is similar for a given average steady-state health as long as the fusion rate is nonzero.

For the passive exchange mechanism in our model that generates asymmetry in 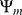, the effectiveness of segregation is determined not only by the fraction of HUs that are exchanged, but also by the total number of HUs per mitochondrion (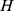), which dictates the discreteness of the health parameter. To keep all other behaviors the same while changing the level of discreteness, we determined the average steady-state health by simultaneously varying 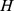 while scaling the number of HUs exchanged during fission. In the absence of fusion, the proportion of exchanged HUs did not affect health, but average steady-state health decreased as 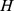 increased (Fig. 9A). This occurred because the distribution of health narrowed (Fig. 9Bi,iv) and hence the replacement of a dysfunctional mitochondrion by a healthy mitochondrion with health 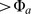 was less effective for health maintenance. Health sharply dropped at 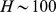; past this amount, the average health was substantially below 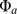 and hence the replication rate was not large enough to compensate for the loss of mitochondria due to autophagy. This underscores the importance of a discrete health variable even in the absence of HU exchange.

The relationship between the total number of HUs and the average steady-state health was more complex when fusion was allowed. Due to the fact that fused mitochondria are unable to undergo autophagy (Methods), higher fusion rates meant that a greater fraction of the mitochondria were temporarily protected from autophagy and hence cells could maintain an average steady-state health just below the autophagy threshold at higher values of 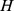. Nevertheless, as 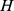 increased, the average steady-state health again declined (Fig. 9A), indicating that the efficacy of HU exchange decreased. Moreover, a sharp drop in health still occurred when the average health dropped below 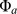 due to the same narrowing of the distribution of health unit values (Fig. 9B). Thus, our simulations indicate that a greater degree of discreteness of mitochondrial health results in higher health due to selective removal of mitochondria associated with the extremes of the distribution of individual healths.

## Discussion

The physical proximity of the mitochondrial DNA (mtDNA), RNA, and proteins to the ROS produced by mitochondria as a side effect of respiration makes them susceptible to damage via chemical interactions. For example, free radicals, including ROS, can cause deletions and base-pair substitutions in DNA [4], potentially rendering proteins encoded by the mtDNA ineffective or even detrimental. The formation of aggregates of misfolded proteins may also exert a toxic effect on the organelles, burdening metabolism [28]. To address cellular capacities for reducing mitochondrial dysfunction, we have developed a model of network dynamics that incorporates mitochondrial motion and spatial heterogeneity in two dimensions. Although this system of interactions between 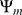, autophagy, fusion, fission, and cytoskeletal-mediated transport is complex (Fig. 1,2), the generality of our model has allowed us to address the relative importance of many potential factors regulating mitochondrial health. In the presence of selective autophagy, the benefits of the generation of 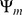 asymmetry during the fusion/fission cycle substantially boost mitochondrial health (Fig. 3,4). During each cycle, the potential to create a healthier and a less healthy mitochondrion from a pair of mitochondria with average health leads to a increase in the average health of the population once autophagy removes the mitochondrion of lower health from the population. In addition, we demonstrated that the optimal operating point for maximal health occurs when the thresholds 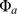 and 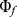 are equal (Fig. 5), suggesting that the molecular mechanism underlying fusion selectivity may act in opposite manners on the autophagy machinery. Indeed, there is evidence that mitochondrial autophagy and fusion are both linked to the transmembrane protein OPA1, suggesting that its concentration may positively and negatively affect fusion and autophagy, respectively [27].

Within the extensive parameter space of our model, we have extracted several simple rules that determine the region of optimal mitochondrial health. Average health is only weakly dependent on density as long as a minimal level of fusion is maintained (Fig. 6). Moreover, imposing a motion dependence on the fusion rate mainly rescaled the effective fusion rate and thereby adjusted health (Fig. 7). These results both suggest that the frequency of fusion events is a relevant metric for evaluating the consequences of perturbations to mitochondrial dynamics on health, and motivate its quantification in future experimental studies. Moreover, the cell may have limits on the absolute number of fusion events, due to the costs of expression for fusion and fission proteins. Our simulations demonstrate that the cell can enhance fusion rates by actively regulating mitochondrial density and motility. Illustrating the importance of autophagy and replication to health maintenance, our simulations also showed that limiting the maximal rate of autophagy to below the damage rate resulted in a sharp decrease in health, while limiting the replication rate caused a decline in population numbers without affecting health (Fig. 8). Where possible, we have used experimentally relevant parameters in our model, and our results are generally robust to changes of large changes of parameters. Moreover, specific details of a given experimental system such as experimentally measured kinetics can be easily incorporated to investigate differences in behavior [29], [30]. Future experiments in a wide range of cell types will help to elucidate which behaviors are universal, for which our model will provide a framework for revealing the underlying mechanisms.

The membrane potential 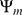 is an experimental readout of the ability of mitochondria to produce ATP. In the interests of simplicity and generality, we have used the health parameter as a proxy for 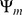, which itself is a function of many components such as the electron transport chain, the number of ATPases utilizing 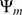, proton leaks across the inner mitochondrial membrane, and other geometric factors [31]. Although the health of a mitochondrion may be directly correlated with 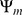, it would be better represented as a discrete variable if it is a function of a discrete underlying quantity affecting 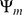, such as the healthy mtDNA copy number [32]. In the absence of an active mechanism for the development of 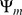 asymmetry, our simulations indicate that the underlying quantity being exchanged during fission must be a unit that exists in small numbers (Fig. 9), such as a mtDNA molecule or a protein aggregate. By condensing “health” into a single parameter, we have systematically analyzed the effects of mitochondrial network dynamics. However, our current model does not address complementarity, the fusion-mediated exchange of different levels of various proteins and solutes between mitochondria, which has also been suggested as another potential benefit of mitochondrial fusion and fission [9]. In future work, this aspect could be explored by increasing the number of parameters that define the health of a mitochondrion.

This study lays the framework for future simulations of the role of mitochondrial network dynamics in the quality control of mitochondrial health. In comparison with previous models [20], [21], which have proven useful for elucidating the systems-level importance of mitochondrial dynamics, we have specifically incorporated the need for spatial proximity and the role of motility in fusion, and thus have the capacity to study how the spatial extent and motility of the mitochondria affect quality control. Furthermore, we have explicitly modeled biogenesis as a separate metabolic process, allowing us to address how population size is stabilized in the presence of mitochondrial turnover. Although we have made simplifying assumptions such as a two-dimensional framework, a square cell geometry, and the absence of intracellular structures such as organelles, our model is easily extended to address more complex cell morphologies and structures that sterically exclude mitochondria. Future work will focus on specific cellular phenomena, such as the spatial heterogeneity of the cytoskeleton and autophagosomes. In addition, our model was implemented to mimic a relatively static cell that maintains a roughly constant number of mitochondria; future investigations will include the increase in mitochondria required for growing and dividing cells. Finally, mitochondrial turnover provides a model for how interactions among the members of a community can enhance collective fitness at the cost of sacrificing individual members, and a computational framework for studying such systems may similarly illuminate key features of system optimality.

## Methods

For each set of parameters (Table 1), we carried out 25 independent simulations to approximate the size of a population of cells and the level of stochasticity present in a typical experimental study of mitochondrial network dynamics [27]. Each simulation represented a cell initialized with 150 mitochondria, an intermediate population size across different mammalian cell types [33]. The mitochondria were initially distributed randomly throughout the cell such that they did not overlap. The autophagy process removes a mitochondrion from the simulation; since fused mitochondria are not subject to autophagy in our simulations, our definition of an individual mitochondrion is the largest possible unit for autophagy, and varying that unit size would be similar to varying the mitochondrial population size or density (Fig. 6). The replication process selects a mitochondrion at random and generates a new mitochondrion with the same health as its progenitor. For the replication rate in Eq. 1, we set 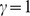; our results were insensitive to changes in 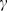. Two strategies were tested for the placement of a new mitochondrion: randomly throughout the cell, or nearby the progenitor. In the latter case, a location within a certain radius of the progenitor is selected at random that does not overlap with other mitochondria. Both strategies converged on quantitatively similar results and hence we used the first strategy for simplicity.

**Table 1:** Typical values of model parameters used in simulations in Fig. 3.

Parameter	Typical value
Simulation time ( 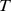 )	10000 min
Time step ( 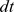 )	1 min
Number of cells	25
Initial number of mitochondria per cell ( 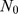 )	150
Cell size	25×25 µm^2^ †
Mitochondrial radius	0.5 µm
Damage rate ( 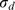 )	5×10^−4^Mito^−1^min^−1^
Fusion rate ( 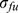 )	0.1Mito^−1^min^−1^ †
Fission rate ( 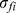 )	0.1Mito^−1^min^−1^
Autophagy rate ( 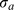 )	3.33×10^−3^Mito^−1^min^−1^
Replication rate ( 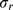 )	0.02Mito^−1^min^−1^
Velocity of transport ( 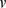 )	0.5 µm min^−1^ †
Autophagy threshold ( 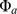 )	0.3†
Fusion threshold ( 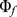 )	0.3†
HUs per Mitochondrion ( 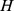 )	10†
HUs exchanged in fission events	2†

† Parameter value was modified as noted in the captions to some figures.

In most simulations, the frequencies of autophagy and replication were determined solely by the number of mitochondria below 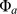 and the total size of the mitochondrial population, respectively. However, for the simulations in Fig. 8, we tracked the cumulative number of autophagy and replication events in each cell and temporarily halted either process when more events had taken place in the preceding three hours than was permitted by the maximum allowed rates. This restriction was only lifted when the running event counter fell below the allowed number, thus preserving the stochastic nature of autophagy and replication while limiting the allowed number of events.

Mitochondrial transport was simulated as a persistent diffusion process in which mitochondria bind to cytoskeletal filaments and move at a constant velocity until they either stochastically unbind from the filament or run into another mitochondrion or the edge of the cell. For simplicity, we ignored any organelles or other spatial features inside the cell, such as the nucleus, that might impede mitochondrial transport; such features would be straightforward to introduce in future extensions of the model. During each time step, each pair of touching mitochondria had the potential for fusion. For most simulations, the probability of fusion for touching mitochondria was 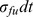. However, in Fig. 7, we also maintained a record of whether the mitochondria came into contact via both, one, or neither being in motion in the previous time step, and scaled the probability by 1, 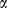, and 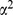, respectively.
